# A genome‐wide association study suggests an association of Chr8p21.3 (GFRA2) with diabetic neuropathic pain

**DOI:** 10.1002/ejp.560

**Published:** 2015-03-18

**Authors:** W. Meng, H.A. Deshmukh, N.R. van Zuydam, Y. Liu, L.A. Donnelly, K. Zhou, Wellcome Trust Case Control Consortium 2 (WTCCC2), Surrogate Markers for Micro‐ and Macro‐Vascular Hard Endpoints for Innovative Diabetes Tools (SUMMIT) Study Group, A.D. Morris, H.M. Colhoun, C.N.A. Palmer, B.H. Smith

**Affiliations:** ^1^ Division of Population Health SciencesMedical Research InstituteNinewells Hospital and School of MedicineUniversity of DundeeUK; ^2^ Centre for Pharmacogenetics and PharmacogenomicsMedical Research InstituteNinewells Hospital and School of MedicineUniversity of DundeeUK; ^3^ Jacqui Wood Cancer CentreMedical Research InstituteNinewells Hospital and School of MedicineUniversity of DundeeUK

## Abstract

**Background:**

Neuropathic pain, caused by a lesion or a disease affecting the somatosensory system, is one of the most common complications in diabetic patients. The purpose of this study is to identify genetic factors contributing to this type of pain in a general diabetic population.

**Method:**

We accessed the Genetics of Diabetes Audit and Research Tayside (GoDARTS) datasets that contain prescription information and monofilament test results for 9439 diabetic patients, among which 6927 diabetic individuals were genotyped by Affymetrix SNP6.0 or Illumina OmniExpress chips. Cases of neuropathic pain were defined as diabetic patients with a prescription history of at least one of five drugs specifically indicated for the treatment of neuropathic pain and in whom monofilament test result was positive for sensory neuropathy in at least one foot. Controls were individuals who did not have a record of receiving any opioid analgesics. Imputation of non‐genotyped SNPs was performed by IMPUTE2, with reference files from 1000 Genomes Phase I datasets.

**Results:**

After data cleaning and relevant exclusions, imputed genotypes of 572 diabetic neuropathic pain cases and 2491 diabetic controls were used in the Fisher's exact test. We identified a cluster in the Chr8p21.3, next to GFRA2 with a lowest *p*‐value of 1.77 × 10^−7^ at rs17428041. The narrow‐sense heritability of this phenotype was 11.00%.

**Conclusion:**

This genome‐wide association study on diabetic neuropathic pain suggests new evidence for the involvement of variants near GFRA2 with the disorder, which needs to be verified in an independent cohort and at the molecular level.

## What's already known about this topic?

- There is currently no published hypothesis‐free genome‐wide association study on neuropathic pain. The genetic contribution of neuropathic pain is poorly understood.

## What does this study add?

- This genome‐wide association study on diabetic neuropathic pain suggests an association of chromosome 8p21.3 with diabetic neuropathic pain. It also provides a calculated narrow‐sense heritability of this trait and confirms that neuropathic pain is a modestly heritable trait.

## 1. Introduction

Neuropathic pain is defined as pain directly caused by a lesion or a disease affecting the somatosensory system (Jensen et al., 2011). Although many common diseases are associated with neuropathic pain (such as herpes zoster), diabetes is one of the most common causes (Belfer and Dai, 2010). Satisfactory relief of neuropathic pain is achieved in less than 30% of these patients, with consequent significant detriment to the quality of life of the remaining individuals (Barrett et al., 2007). In addition, the disorder represents a significant economic burden to health‐care systems (Tarride et al., 2006; Dworkin et al., 2010).

Epidemiological studies have proposed multiple risk factors associated with neuropathic pain from cross‐sectional studies, including older age, female gender, manual occupation, lower educational attainment, living in a rural area or poor accommodation (Torrance et al., 2006; Smith et al., 2007). Additional risk factors for diabetic neuropathic pain have been proposed, including smoking, hypertension, obesity, hypercholesterolaemia and duration of diabetes (Tesfaye et al., 2005; Jensen et al., 2006). Epidemiological studies can identify risk factors and effective preventive strategies in parallel with the search for the underlying causative biological mechanisms, such as genetic pathways (Smith et al., 2007).

Understanding the genetic factors associated with neuropathic pain would assist in identifying the underlying causal mechanisms and potentially indicate molecular targets for pharmacological research. Animal models have been widely applied in genetic research in neuropathic pain. The heritability of neuropathic pain was estimated to be around 30% in rat models (Devor et al., 2005). Global gene expression changes were observed in dorsal root ganglions and the spinal cord in the spinal nerve ligation model of neuropathic pain using rats (Wang et al., 2002). These genes include immediate early genes; genes encoding ion channels and signalling molecules that contribute to the excitability of neurons; and genes that are indicative of secondary events such as neuroinflammation. Chessell et al. (2005) reported that *P2X7* purinoceptor gene is essential for neuropathic pain. Trang et al. (2009) proposed that *P2X4* receptors in the rats' microglia cells activated by peripheral nerve injury lead to neuropathic pain via the release of brain‐derived neurotrophic factor. Other studies in mouse models reported that *TLR4* and *CACNG2* genes are involved in neuropathic pain (Nissenbaum et al., 2010; Wang et al., 2013). Several candidate genes for neuropathic pain therefore exist, although none has been firmly confirmed or replicated in further human studies. Recent family studies and twin studies have found important genetic factors involved in pain perception in humans (Norbury et al., 2007). Genome‐wide association study (GWAS) is a useful and efficient method to identify potential candidate genes for common complex disorders using DNA chips (McCarthy et al., 2008). The DNA chips can genotype hundreds of thousands of single nucleotide polymorphisms (SNP) in individuals, comparing variants between cases and controls. So far, no GWAS has been performed specifically on neuropathic pain.

To identify the genetic factors associated with neuropathic pain in diabetes, we performed this GWAS using a UK‐based diabetic population.

## 2. Methods

### 2.1 Participants

We used the datasets from the Genetics of Diabetes Audit and Research Tayside (GoDARTS) project in this study. The project recruits consented patients with type 2 diabetes and non‐diabetic matching controls throughout Tayside, Scotland, to identify genetic factors related to diabetes, including susceptibility, complications and response to treatment. Participants attend for a simple baseline clinical examination and complete a lifestyle questionnaire as well as provide blood and urine samples. The consent provided by participants at the time of recruitment not only allows the use of their data and samples (including extracted DNA) for research purposes but also allows the data to be linked anonymously to datasets derived from patients' medical records. These datasets include prescribing data, hospital admissions, outpatient appointments and Scottish Care Information‐Diabetes Collaboration (SCI‐DC) – an electronic health record used by health‐care professionals throughout Scotland for the care of patients with diabetes. Further information, including data access procedures, is available at http://diabetesgenetics.dundee.ac.uk/. The GoDARTS study has been approved by Tayside Committee on Medical Research Ethics and informed consent was obtained from all patients (REC reference 053/04).

So far, the project had recruited 9439 patients and 6927 of them had been genotyped. For this study, we examined GoDARTS data derived from linked records on routine health care on participants' prescription history from the date of recruitment to June 2011, and monofilament testing results for the presence of peripheral sensory neuropathy, as well as directly provided data on age, gender and body mass index (BMI). The monofilament test is a simple neurological test carried out annually on diabetic patients to check peripheral sensation. A monofilament is pressed at various sites on both feet with approximately 10 g of pressure for a short time (2 s) (Booth, 2000). Absence of sensation in at least two out of five sites in one foot is a positive test, considered indicative of likely peripheral neuropathy (Booth, 2000).

### 2.2 Definition of neuropathic pain cases and controls

A neuropathic pain case was defined in this study as a type 2 diabetic individual with a history of at least one prescription of any of the following five medicines, which are effective and recommended in diabetic peripheral neuropathy (Attal et al., 2010; Finnerup et al., 2010; NICE, 2013) and used less frequently for other indications: duloxetine, gabapentin, pregabalin, capsaicin cream/patch and lidocaine patch. The cases also had positive monofilament tests in at least one foot, indicating the likely presence of sensory neuropathy.

A control was defined as a type 2 diabetic individual with no prescription history of these five drugs, nor of the following 16 opioid analgesics (buprenorphine, codeine phosphate, diamorphine, dihydrocodeine, dipipanone, fentanyl, hydromorphone, meptazinol, methadone, morphine, oxycodone, papaveretum, pentazocine, pethidine, tapentadol and tramadol). Individuals with a prescription history of amitriptyline, carbamazepine or nortriptyline were excluded from controls since these are also frequently used to treat other disorders (although these drugs are effective in neuropathic pain), and the clinical information available from GoDARTS included neither the indication for prescribing nor the presence of these co‐morbidities.

### 2.3 Genotyping and quality control

The GoDARTS diabetic individuals were genotyped by either Affymetrix SNP6.0 chips (3673 patients) funded by the Wellcome Trust Case Control Consortium 2 (WTCCC2) project (GoDARTS and UKPDS et al., 2011), or by Illumina OmniExpress chips (3254 patients) funded by the Surrogate markers for Micro‐ and Macro‐vascular hard endpoints for Innovative diabetes Tools (SUMMIT) project (Fagerholm et al., 2012). Genotype data quality controls were undertaken using the protocols that were established for the WTCCC2 studies (GoDARTS and UKPDS et al., 2011) and the SUMMIT studies (Fagerholm et al., 2012).

### 2.4 Statistical analysis

SHAPEIT and IMPUTE2 were used for imputation of non‐genotyped SNPs in the Affymetrix SNP6.0 chips and Illumina OmniExpress chips using reference files from the 1000 genome phase I datasets (Howie et al., 2009; Delaneau et al., 2011). IMPUTE2 uses an r^2^ score to evaluate the quality of a specific imputed genotype. We used the recommended r^2^ > 0.3 to filter out badly imputed SNPs. PLINK was the main software for data manipulation, and routine quality control steps were frequently applied during analyses (removing SNPs with over 10% genotyping missing, or with minor allele frequency less than 1%, or those that failed Hardy–Weinberg tests *p* < 0.00001, and removing individuals with more than 10% genotype data missing) (Purcell et al., 2007). SNPs on the X and Y chromosomes and mitochondrial SNPs were excluded from analyses. Population stratification analysis was based upon multidimensional scaling integrated in PLINK to detect any different ancestry in the cohort, with a lambda value indicating the level of stratification. For good quality datasets with minimum ancestry mixture, lambda value should be close to 1. Removal of related samples was based upon pi‐hat >0.10 in PLINK. The *p*‐values for SNP associations were generated based upon Fisher's exact test integrated in PLINK. A *p*‐value of less than 10^−6^ was considered to be suggestive of an association, warranting further exploration. SNPnexus was applied for SNP functional annotation and HaploView was used for generating Manhattan plots (Barrett et al., 2005; Dayem Ullah et al., 2013). LocusZoom was used for regional visualization (Pruim et al., 2010). The corresponding Q‐Q plot, a tool used to evaluate differences between cases and controls caused by potential confounders (different genotyping laboratories, different DNA extraction methods, etc.), was generated by SNPEVG (Wang et al., 2012). The whole workflow was shown in Supporting Information Fig. S1. Narrow‐sense heritability was calculated by restricted maximum likelihood analysis based upon common SNPs in both chips using GCTA, a tool for genome‐wide complex trait analysis (Lee et al., 2011). Narrow‐sense heritability is defined as the ratio of total phenotypic variance that is due to additive genetic effects (Lee et al., 2011). Means of age, gender and BMI were compared between cases and controls using independent *t*‐test in SPSS 21 (IBM Corp, Armonk, NY, USA).

## 3. Results

**Figure 1:**
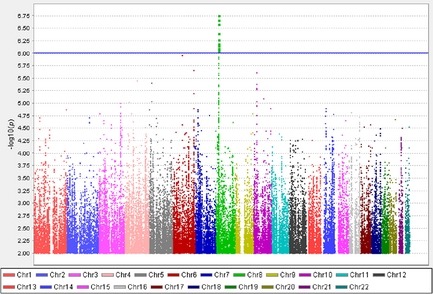
Manhattan plot of the genome‐wide association study on neuropathic pain using imputed single nucleotide polymorphisms. *X*‐axis represents 22 autosomes. *Y*‐axis means the −log 10 of *p*‐values. The blue line is the cut‐off *p*‐value of 10^−6^.

In this general diabetic population, we identified 970 unrelated patients with a prescription history of one or more of the five relevant drugs, representing 14.41% of the whole genotyped diabetic population. Of these, 572 individuals (297 males and 275 females) had positive monofilament test results in at least one foot, making up 8.50% (572/6927) of the total genotyped diabetic population. Among these cases, 249 samples were genotyped on an Illumina platform and 323 samples were genotyped on an Affymetrix platform. Of the remaining 5957 individuals, 310 individuals were removed either because they were outlier in the population stratification analysis or because they were related to another sample. Among the rest, 2666 were identified as receiving 1 of the 16 opioid analgesics, and a further 490 individuals were excluded since they had a prescription history of amitriptyline, carbamazepine or nortriptyline. Thus, we identified and included 2491 controls, including 1503 males and 988 females. Cases therefore represented 18.7% of the eligible cohort (572/572 + 2491). Among these controls, 1244 were genotyped on the Illumina platform and 1247 were genotyped on the Affymetrix platform. The average (mean ± standard deviation) age and BMI in cases were 66.82 ± 10.69 and 33.28 ± 6.20, respectively. The average age and BMI in controls were 66.86 ± 10.25 and 34.99 ± 6.98, respectively. There was no statistical difference in age between cases and controls, but the differences in BMI and gender were statistically significant (*p* < 0.01) (Supporting Information Table S1). Altogether, 6,494,962 imputed SNPs survived from routine quality control checking and imputation quality r^2^ > 0.3. Since the multidimensional scaling analysis for population stratification found a lambda of 1.014 for the cleaned datasets, no further adjustment based upon population stratification was applied (Supporting Information Fig. S2). The corresponding Q‐Q plot is shown in Supporting Information Fig. S3. Using Fisher's exact test, there was a cluster appearing in the Manhattan plot (only SNPs with *p*‐values less than 0.01 were used to generate the plot). Although none of the SNPs reached genome‐wide significance (5 × 10^−8^), the cluster in chromosome 8p21.3 (Chr8p21.3), next to *GFRA2* gene, still indicated possible associations (Fig. 1). The top SNP in this region was rs17428041, with a lowest *p*‐value of 1.77 × 10^−7^ and an odds ratio (OR) of 0.67 (95% confidence interval: 0.57–0.78). Table 1 summarizes the significant SNPs found in the region. Supporting Information Fig. S4 shows the regional plot of the identified loci. The heritability of neuropathic pain was estimated to be 11.00% in this diabetic population. Since BMI and gender were statistically different between cases and controls, a logistic regression analysis adjusting for these factors was performed. An extra peak was found in the chromosome 12p13 (Supporting Information Fig. S5). The *p*‐value of the top SNP (rs11615866) is 1.08 × 10^−6^ and all the significant SNPs in the chr8p21.3 and chr12p13 are summarized in the Supporting Information Table S2.

**Table 1:** Significant SNPs in Chr8p21.3 next to GFRA2

Chr	SNP	Position	Gene	Minor allele	Allele frequency in cases (%)	Allele frequency in controls (%)	*p*‐value	OR	Information about the SNP
8	rs4872521	21707713	Intergenic	G	21.53	28.82	5.40 × 10^−7^	0.68	Imputed
8	rs4872522	21707844	Intergenic	C	21.53	28.78	6.47 × 10^−7^	0.68	Imputed
8	rs10098807	21708824	Intergenic	A	21.63	28.84	7.00 × 10^−7^	0.68	Imputed
8	rs11774105	21710146	Intergenic	C	21.72	29.06	4.03 × 10^−7^	0.68	Imputed
8	rs17428041	21711431	Intergenic	C	21.53	29.08	1.77 × 10^−7^	0.67	In the Illumina OmniExpress
8	rs17615364	21711580	Intergenic	A	21.58	29.08	2.20 × 10^−7^	0.67	Imputed
8	rs11776842	21711651	Intergenic	C	21.58	29.08	2.20 × 10^−7^	0.67	Imputed
8	rs12545534	21712401	Intergenic	A	21.58	29.02	2.62 × 10^−7^	0.67	In the Illumina OmniExpress
8	rs11780601	21717841	Intergenic	T	18.79	25.63	7.98 × 10^−7^	0.67	In the Illumina OmniExpress

*p*‐values and ORs were calculated using Fisher's exact test. Chr, chromosome; SNP, single nucleotide polymorphisms; OR, odds ratio.

## 4. Discussion

This GWAS on neuropathic pain is based upon a well‐defined diabetic population in the United Kingdom, using a pragmatic method of case definition and ascertainment, and found a locus that may be associated with painful diabetic neuropathy.

The assessment of neuropathic pain has been internationally standardized for primary care and specialist settings (Haanpää et al., 2011; Jones and Backonja, 2013). However, these detailed assessment methods are not suitable for population‐based settings where thousands of patients are to be phenotyped. Although brief screening instruments, aimed at detecting pain with neuropathic characteristics, have been used in population‐based research, they are imperfect and have not been validated in general population settings (Haanpää et al., 2011). Therefore, there is no practically applicable neuropathic pain gold standard phenotype for large human studies. No formal assessment of (neuropathic) pain was made in the GoDARTS cohort. An appropriate case definition of neuropathic pain in a general population cohort is difficult to determine and there is currently no consensus on this among researchers. A good phenotype definition will cluster relatively homogeneous individuals with similar clinical conditions. For genetic association studies in particular, the wrong phenotype can lead to false‐positive and false‐negative results (Belfer and Dai, 2010). In our study, to achieve a relatively homogeneous and specific case population, we based the case definition on a history of receiving drugs that are mostly used only for neuropathic pain and on recorded evidence of peripheral neuropathy, as shown by responses to the monofilament test. This allowed us to have a more homogeneous case population of diabetic neuropathic pain at the cost of decreased case numbers. To achieve a homogeneous control population, we removed diabetic individuals with a history of using opioid analgesics. Any individuals using drugs that are frequently used for treating both neuropathic pain and other disorders were also excluded from controls. While we recognize that other drugs (particularly tricyclic antidepressants) are used in the treatment of neuropathic pain, these are also frequently used for other indications (mainly depression), and we therefore did not include individuals identified only on receipt of these drugs as cases, to optimize homogeneity. Similarly, the drugs we used for case definition can be used for other indications. In particular, duloxetine is indicated for depression, and depression is relatively common in diabetes. However, it is not a first‐line treatment for depression, whereas it is recommended as a first‐line treatment for diabetic neuropathy (NICE, 2013). We therefore decided to include it in our case definition. Had we excluded those receiving duloxetine, we would only have identified 516 cases, and our study would have been more under‐powered. Similarly, it has been demonstrated in the United Kingdom that patients in primary care with neuropathic pain are sometimes not prescribed with the specific medications of known effectiveness (Torrance et al., 2013, 2007; Hall et al., 2008). Therefore, the controls in our study might have untreated neuropathic pain, again diluting the results. Lack of available data on pain status precludes assessment of this effect. Methods of selecting homogeneous samples in population‐based GWAS have been attempted in other common disorders (Meng et al., 2012a, 2012b). In our study, which had a mean age of 66.83 ± 10.61, 18.7% of eligible participants were defined as cases. This is similar to the proportion identified in a large community‐based UK study of diabetes of similar mean age (63.60 ± 11.80), which found that 21% had both neuropathy and positive responses to a validated neuropathic pain symptom score (Abbott et al., 2011). Furthermore, we found a similar gender distribution, with a female : male ratio of 1.32, compared with 1.21 (Abbott et al., 2011), and this tends to support the validity of our phenotype.

The significant SNP cluster was identified in Chr8p21.3, with a lowest *p*‐value of 1.77 × 10^−7^ at rs17428041, spanning 10 kb from position 21707713 to position 21717841. The OR is 0.67 per copy of the C allele of rs17428041, suggesting that this allele is protective and that individuals with an additional C allele in this SNP will have only 0.67 odds of being a case compared to those with a T allele. The locus is next to *GFRA2* gene. *GFRA2* encodes a glycosylphosphatidylinositol (GPI)‐linked cell surface receptor for both glial cell line‐derived neurotrophic factor (GDNF) and neurturin (NTN), but preferentially for NTN (Jing et al., 1997). GDNF and NTN are two structurally related, potent neurotrophic factors that are involved in the control of neuron survival and differentiation (Baudet et al., 2000). GDNF participates in the modulation of nociceptive signals especially during neuropathic pain states (Dong et al., 2005). Furthermore, exogenous GDNF resulted in the relief of pain in different neuropathic pain rat models (Boucher et al., 2000). In pancreatic cancer, which is closely linked with neuropathic pain, NTN has been shown to be produced by cancer cells, and to increase the cells' biological properties, trigger neuroplastic alterations, neural invasion and influence pain sensation via the *GFRA2* receptor (Wang et al., 2014). It was the *GFRA2* receptor that mediated the pro‐algesic effect of the NTN/*GFRA2* axis via the corresponding nociceptors (Wang et al., 2014). NTN and *GFRA2* were highly unregulated, especially in intrapancreatic nerves and the extracellular matrix (Wang et al., 2014). In a formalin test, the GFRA2 knockout mice showed a markedly attenuated persistent phase response to stimuli, suggesting a deficit in inflammatory pain responses (Lindfors et al., 2006).

There were no sporadic SNPs passing *p*‐value less than 10^−6^. Although a *p*‐value of 5 × 10^−8^ is often accepted as the threshold for GWAS significance, this might be too stringent (Do et al., 2014). No previous evidence has been published linking the *DOK2* gene (next to the cluster in Chr8p21.3 from the opposite direction) with any pain mechanisms. Therefore, we have not further explored possible relationships between the identified SNPs and *DOK2* with neuropathic pain. Narrow‐sense heritability of diabetic neuropathic pain was estimated to be 11.00% in this diabetic population. This estimate excludes the contribution of gene–gene interactions, gene–environment interactions, etc., so the actual heritability of this phenotype is likely to be larger. This is the first report of the heritability of neuropathic pain in humans, although heritability has been demonstrated in rat models and in other pain conditions in humans (Devor et al., 2005; Hocking et al., 2012). Although our heritability was relatively low in comparison with other pain conditions, we have suggested that neuropathic pain is a heritable trait and further genetic research is warranted.

We had moderated power in this study due to the limited number of cases. According to CaTS, using a multiplicative model, we had 80% power to detect a genotypic relative risk of 1.44 (or 0.69) for variants with a minor allele frequency of 30% when the disease prevalence in the population is 10% and the significant level is 10^−6^ (Skol et al., 2006). However, development and application of new criteria in selecting the maximum number of homogeneous cases will enhance each individual SNP's relative risk value when evaluating power (Belfer and Dai, 2010). It is important that such criteria are agreed internationally, to allow future studies to replicate findings directly, in different settings. A new and valid phenotyping approach to neuropathic pain will not only improve data and study quality but also help us to discover novel mechanisms of pain at a molecular level. It has the potential for identifying drug targets and eventually leading to better therapeutic management. The monofilament test is a simple and inexpensive screening tool for identifying diabetic peripheral neuropathy in clinical settings (Lee et al., 2003), although the accuracy has been challenged (Dros et al., 2009).

Although there is no other published GWAS on neuropathic pain, a GWAS study on chronic widespread pain has identified that a locus at Chr5p15.2 between *CCT5* and *FAM173B* might be associated with the disorder (Peters et al., 2013). Another GWAS study reported that the C allele of rs2952768 in the Chr2q33.3 was associated with more analgesic requirements in human (Nishizawa et al., 2014). Multiple GWAS studies have proposed genes (*PRDM16*, *TRPM8* or *LRP1*) and locus (Chr8q22.1) to be involved in the migraine (Anttila et al, 2010; Chasman et al., 2011). Therefore, there is growing evidence of the involvement of SNPs in pain pathways, although much more research is required, including particularly replication studies, and consensus on feasible and relevant phenotype ascertainment. None of the SNPs in above‐mentioned loci was positive in our study.

## 5. Conclusion

The analysis provided support that SNPs next to *GFRA2* in the Chr8p21.3 may be associated with neuropathic pain in diabetes. We used a new approach in this study to define neuropathic pain cases, based upon routine prescribing data and evidence of neuropathy, to achieve a reasonably homogeneous phenotype. Our next step is to attempt replication of significant SNPs in independent cohorts and focus upon the molecular mechanisms that may be responsible for the association signals. The findings of these studies will confirm hypothesized pathways of pain mechanisms or suggest new ones, and provide possible drug targets for pain treatment, with potential patient benefit.

## Author contributions

W.M. analysed the data and prepared the manuscript. H.A.D. contributed to the manuscript preparation. N.R.Z., Y.L., L.A.D. and K.Z. contributed to the analysis of the GoDARTS datasets. A.D.M. and H.M.C. reviewed the manuscript and contributed to the discussion. C.N.A.P. made a significant contribution to the discussion. B.H.S. supervised the overall pain project and contributed significantly to the manuscript.

## Supporting information


**Figure S1.** Workflow of the GWAS on neuropathic pain.

**Figure S2.** Multidimensional scaling analysis by PLINK to detect population stratification.

**Figure S3.** Q‐Q plot compared expected and observed log 10(1/*p*) values.

**Figure S4.** Regional plot of Chr8p21.3 r^2^ represents the level of linkage disequilibrium of SNPs.

**Figure S5.** Manhattan plot of the GWAS on neuropathic pain using logistic regression with age, gender, body mass index adjusted. *X*‐axis represents 22 autosomes. *Y*‐axis means the −log 10 of *p*‐values. The blue line is the cut‐off *p*‐value of 10^−5^.

**Table S1.** Information on covariates between cases and controls. Age and BMI (body mass index) are presented as mean + standard deviation.

**Table S2.** Significant SNPs in the Chr8p21.3 and Chr12p13 loci.
