# Supplementary figures and images for: A genome‐wide association study suggests an association of Chr8p21.3 (GFRA2) with diabetic neuropathic pain
Source: Eur J Pain. 2015 Mar 18;19(3):392–9. doi: 10.1002/ejp.560 (PMC4737240; doi:10.1002/ejp.560)

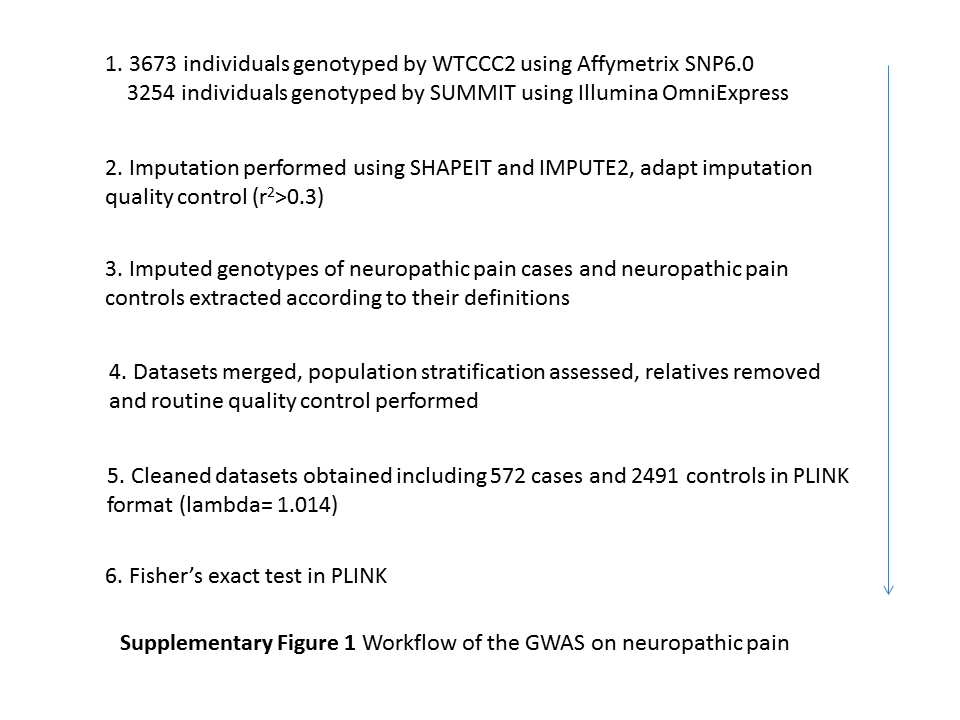

Supplement: Supplementary file 1 — Figure S1. Workflow of the GWAS on neuropathic pain. [file EJP-19-392-s001.tif]

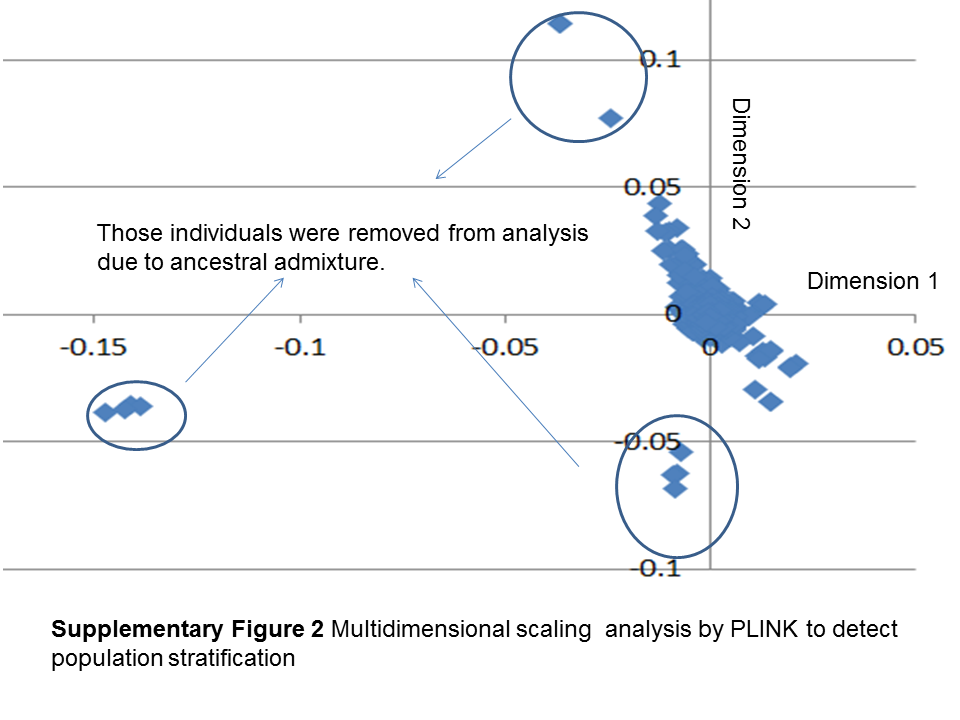

Supplement: Supplementary file 2 — Figure S2. Multidimensional scaling analysis by PLINK to detect population stratification. [file EJP-19-392-s002.tif]

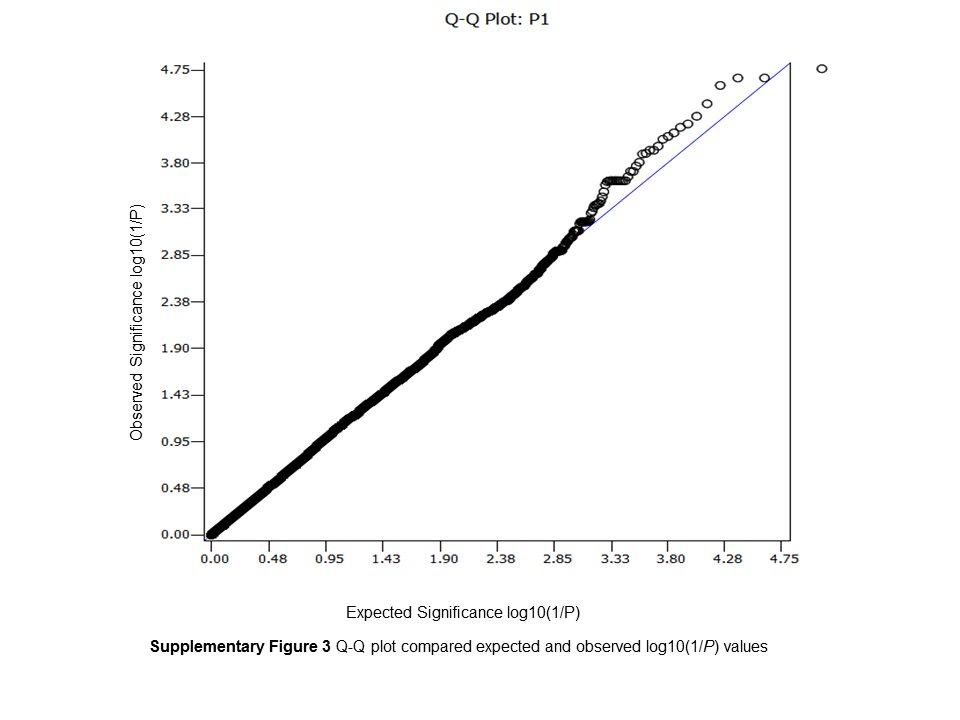

Supplement: Supplementary file 3 — Figure S3. Q‐Q plot compared expected and observed log 10(1/p) values. [file EJP-19-392-s003.tif]

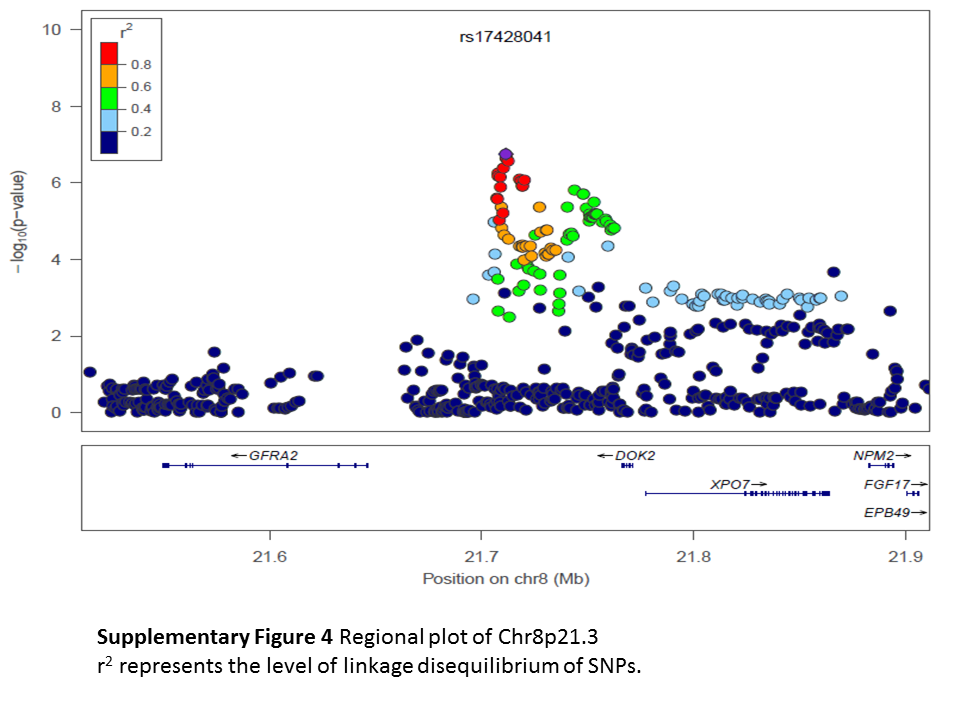

Supplement: Supplementary file 4 — Figure S4. Regional plot of Chr8p21.3 r2 represents the level of linkage disequilibrium of SNPs. [file EJP-19-392-s004.tif]

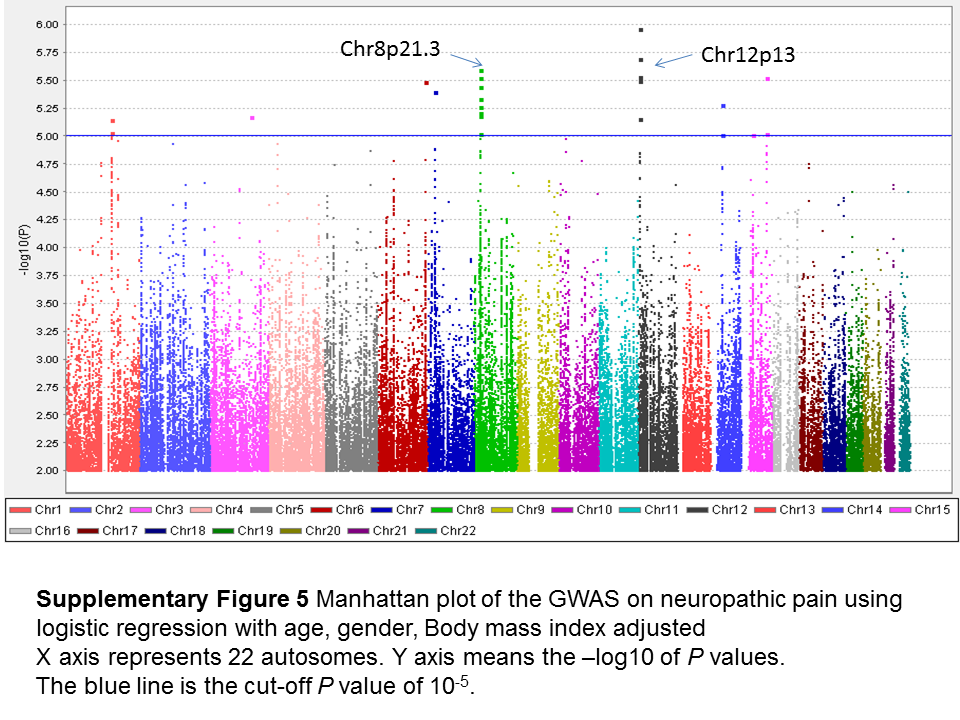

Supplement: Supplementary file 5 — Figure S5. Manhattan plot of the GWAS on neuropathic pain using logistic regression with age, gender, body mass index adjusted. X‐axis represents 22 autosomes. Y‐axis means the −log 10 of p‐values. The blue line is the cut‐off p‐value of 10−5. [file EJP-19-392-s005.tif]
