# Supplementary material for: A genome‐wide association study suggests an association of Chr8p21.3 (GFRA2) with diabetic neuropathic pain
Source: Eur J Pain. 2015 Mar 18;19(3):392–9. doi: 10.1002/ejp.560 (PMC4737240; doi:10.1002/ejp.560)
Supplement: Supplementary file 6 — Table S1. Information on covariates between cases and controls. Age and BMI (body mass index) are presented as mean + standard deviation. [file EJP-19-392-s006.doc]

|  | Age | Gender(male/female) | BMI |
| --- | --- | --- | --- |
| Cases | 66.82+10.69 | 297/275 | 33.28+6.20 |
| Controls | 66.86+10.25 | 1503/988 | 34.99+6.98 |
| *P* value | *P* > 0.05 | *P* < 0.01 | *P* < 0.01 |

**Supplementary Table 1** Information on covariates between cases and controls

Age and BMI (body mass index) are presented in as mean+standard deviation
