# Supplementary material for: A genome‐wide association study suggests an association of Chr8p21.3 (GFRA2) with diabetic neuropathic pain
Source: Eur J Pain. 2015 Mar 18;19(3):392–9. doi: 10.1002/ejp.560 (PMC4737240; doi:10.1002/ejp.560)
Supplement: Supplementary file 7 — Table S2. Significant SNPs in the Chr8p21.3 and Chr12p13 loci. [file EJP-19-392-s007.doc]

| Chr | SNP | Position | Gene | Minor allele | Allele frequency in cases (%) | Allele frequency in controls (%) | *P* value | OR | Information about the SNP |
| --- | --- | --- | --- | --- | --- | --- | --- | --- | --- |
| 8 | rs4872521 | 21707713 | Intergenic | G | 21.53 | 28.82 | 5.49X10^-6^ | 0.68 | Imputed |
| 8 | rs4872522 | 21707844 | Intergenic | C | 21.53 | 28.78 | 6.15X10^-6^ | 0.68 | Imputed |
| 8 | rs10098807 | 21708824 | Intergenic | A | 21.63 | 28.84 | 6.55X10^-6^ | 0.68 | Imputed |
| 8 | rs11774105 | 21710146 | Intergenic | C | 21.72 | 29.06 | 4.59X10^-6^ | 0.68 | Imputed |
| 8 | rs17428041 | 21711431 | Intergenic | C | 21.53 | 29.08 | 2.54X10^-6^ | 0.67 | In the Illumina OmniExpress |
| 8 | rs17615364 | 21711580 | Intergenic | A | 21.58 | 29.08 | 3.00X10^-6^ | 0.67 | Imputed |
| 8 | rs11776842 | 21711651 | Intergenic | C | 21.58 | 29.08 | 3.00X10^-6^ | 0.67 | Imputed |
| 8 | rs12545534 | 21712401 | Intergenic | A | 21.58 | 29.02 | 3.57X10^-6^ | 0.67 | In the Illumina OmniExpress |
| 8 | rs11780601 | 21717841 | Intergenic | T | 18.79 | 25.63 | 9.47X10^-6^ | 0.67 | In the Illumina OmniExpress |
| 12 | rs10492090 | 5391393 | Intergenic | A | 15.91 | 12.05 | 2.03X10^-6^ | 2.19 | Genotyped by both chips |
| 12 | rs11615866 | 5393329 | Intergenic | T | 16.03 | 12.00 | 1.08X10^-6^ | 2.26 | Imputed |
| 12 | rs16933383 | 5400620 | RP11-1038A11.3 | T | 16.31 | 12.76 | 3.03X10^-6^ | 2.03 | in the Illumina OmniExpress |
| 12 | rs16933389 | 5401196 | RP11-1038A11.3 | G | 16.46 | 12.89 | 3.02X10^-6^ | 2.03 | Imputed |
| 12 | rs7979058 | 5401450 | RP11-1038A11.3 | G | 16.34 | 12.81 | 3.18X10^-6^ | 2.03 | Imputed |
| 12 | rs7137245 | 5402869 | RP11-1038A11.3 | C | 16.46 | 12.90 | 3.04X10^-6^ | 2.03 | Imputed |
| 12 | rs11063602 | 5405457 | RP11-1038A11.3 | T | 16.37 | 12.86 | 2.94X10^-6^ | 2.03 | Imputed |

**Supplementary Table 2** Significant SNPs in in the Chr8p21.3 and Chr12p13 loci

Chr: chromosome, SNP: single nucleotide polymorphisms, OR: odds ratio.

*P* values and ORs were calculated based on an additive model in the logistic regression analysis with covariates of age, gender and body mass index (BMI).

Most of the SNPs (5 out of 7) are in a large intervening non-coding RNA (lincRNA) transcript, RP11-1038A11.3. The functions of the lincRNA transcript are not clear. The reason that Chr12p13 becomes a peak is mainly due to the adjusting of the gender and BMI difference between cases and controls. The *P* values of the SNPs in the Chr8p21.3 were increased after adjusting covariates while the locus still remains to be a clear peak in the plot.
